# The Operative Treatment of Chronic Glaucoma

**Published:** 1893-03

**Authors:** 


THE OPERATIVE TREATMENT OF CHRONIC GLAUCOMA.—Collins (Roy. Lond. Ophth. Hosp. Rep., xiii, 2) infers that an iridectomy may remedy the increased tension due to apposition of the root of the iris to the posterior surface of the cornea in the following ways: 1. When this apposition is recent, very slight means are sometimes sufficient, the escape of the aqueous and a drag on the iris being enough. 2. In some recent and acute cases the iris tears away from its extreme root, thus leaving a large portion of the filtration area free for drainage, even if the remainder of the iris retains its faulty position. 3. In some cases a permanent gap is maintained in the walls of the globe by the prolapse of a fold of the iris in the wound. This iris tissue subsequently either becomes stretched and atrophied or ruptures periodically, thus allowing the aqueous to pass through it into the subconjunctival tissue, and become absorbed by the lymphatics and vessels. By these means a new channel of exit for the aqueous is formed. He emphasizes the advisability of performing iridectomy for chronic glaucoma in the early stages of the disease, before the apposition of the root of the iris to the cornea has resulted in adhesion.—*New York Medical Journal.*

